# Transarterial embolization with n-butyl-2-cyanoacrylate for a ruptured aneurysm at the artery of Davidoff and Schechter

**DOI:** 10.1016/j.radcr.2024.02.063

**Published:** 2024-03-21

**Authors:** Kei Yanai, Genta Fujii, Shinji Kawamura, Ako Matsuhashi, Kazuaki Naemura, Satoshi Koizumi, Gakushi Yoshikawa

**Affiliations:** a Department of Neurosurgery, Showa General Hospital, Tokyo, Japan; b Department of Neurosurgery, Tokyo Metropolitan Tama Medical Center, Tokyo, Japan; c Department of Neurosurgery, Tokyo Metropolitan Caner and Infectious Disease Komagome Hospital, Tokyo, Japan; d Department of Neurosurgery, Tokyo Shinjuku Medical Hospital, Tokyo, Japan; e Department of Neurosurgery, Mitsui Memorial Hospital, Tokyo, Japan; f Department of Neurosurgery, The University of Tokyo Hospital, Tokyo, Japan

**Keywords:** Artery of Davidoff and Schechter, Arteriovenous fistula, N-butyl-2-cyanoacrylate, Ruptured aneurysm

## Abstract

The artery of Davidoff and Schechter (ADS) is mostly identified in pathological conditions such as dural arteriovenous fistulas and brain tumors. Herein, we report a rare case of a ruptured aneurysm of the ADS, which was one of the feeders of a falcotentorial dural arteriovenous fistula. We performed endovascular embolization of the aneurysm and parent artery using n-butyl-2-cyanoacrylate. Complete occlusion of the fistula was achieved by another feeder after the acute phase. To our best knowledge, only a few reports on embolization of ruptured ADS aneurysms exist. Furthermore, this is the first report on the embolization of a ruptured ADS aneurysm using n-butyl-2-cyanoacrylate. This case highlights that endovascular n-butyl-2-cyanoacrylate embolization could be a useful treatment for a ruptured ADS aneurysm.

## Introduction

Tentorial dural arteriovenous fistula (dAVF) is uncommon, accounting for approximately 5% of all dAVF cases [1]. In some cases of tentorial dAVF, the artery of Davidoff and Schechter (ADS), a branch of the posterior cerebral artery (PCA) supplying the cerebellar tentorium, has also been reported as a feeder [2]. We present a case of subarachnoid hemorrhage (SAH) caused by an aneurysm of the ADS. The aneurysm was successfully embolized using N–butyl-2-cyanoacrylate (NBCA). Our case report highlights the usefulness of NBCA embolization for this rare pathology.

## Case report

**Fig. 1:**
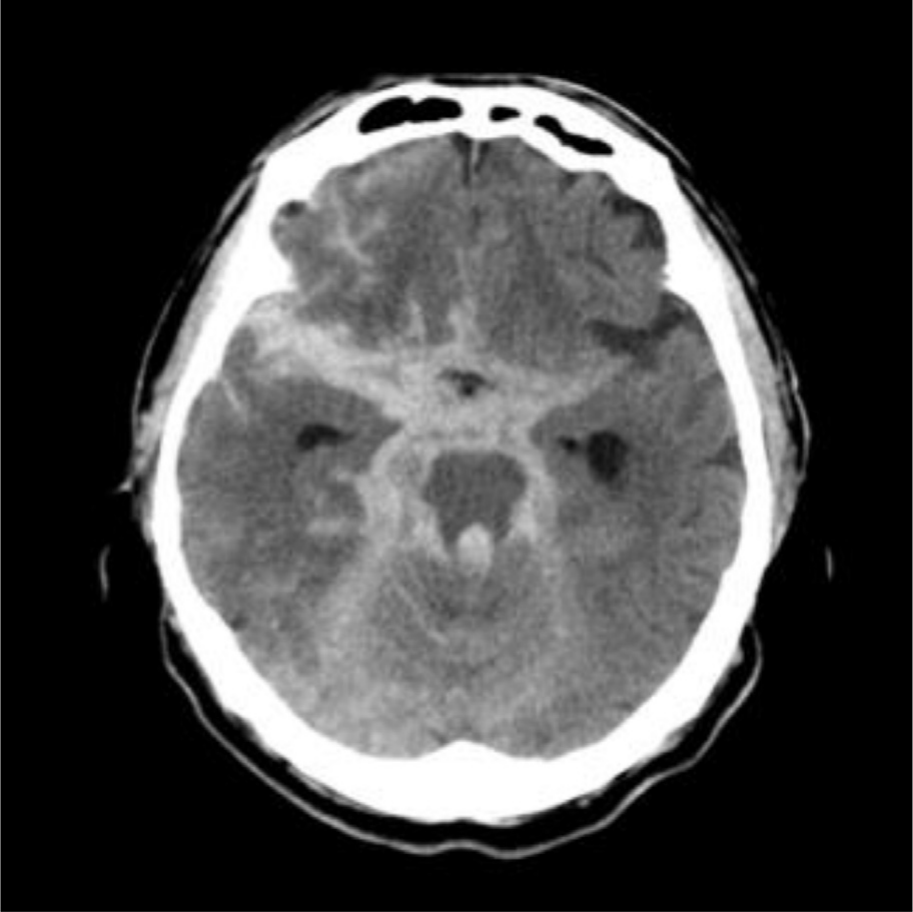
Computed tomography findings in an older patient with subarachnoid hemorrhage. The computed tomogram shows a thick subarachnoid hemorrhage, predominantly in the right ambient cistern.

A 79-year-old man without a specific past medical history was transferred to our hospital due to poor alertness. Upon admission, the patient showed a Glasgow Coma Scale (GCS) score of 9 (eye 4; verbal 2; and motor 3; [GCS E4V2M3]) and left conjugate deviation. Subsequently, a head computed tomography (CT) detected a thick SAH in the posterior fossa (Fig. 1).

**Fig. 2:**
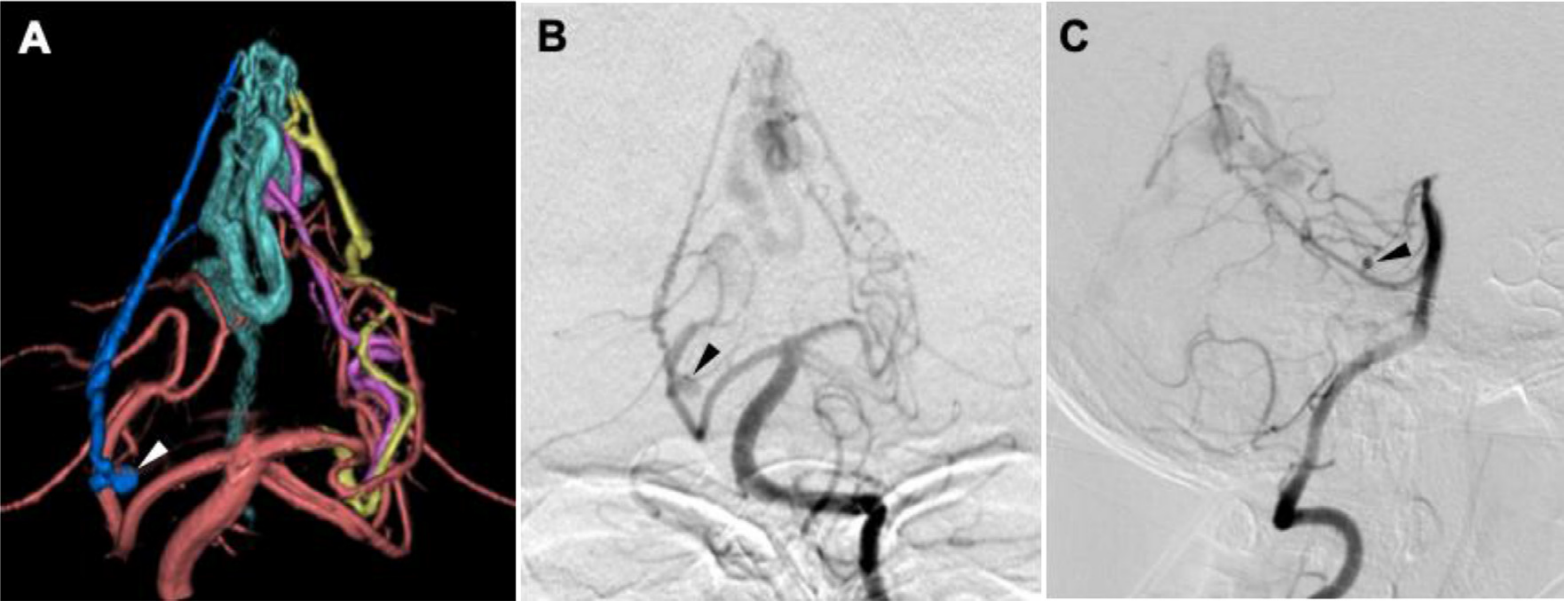
Three-dimensional rotational angiogram (A) of the vertebral artery. The deep-blue vessel is the right artery of Davidoff and Schechter (ADS) with an aneurysm (arrowhead). The yellow and purple marked vessels are the left ADSs. Frontal (B) and lateral (C) views of angiograms of the vertebral artery show a falcotentorial dural arteriovenous fistula fed by the right ADS with an aneurysm (arrowhead), and two left ADS feeders draining into the superior vermian vein (Cyan vessel in A).

The cerebral angiogram revealed a falcotentorial dAVF. The fistula was fed by the bilateral ADSs, middle meningeal arteries (MMAs), superficial temporal arteries, and occipital arteries. The fistula was located at the falcotentorial point and drained into the marginal sinus through the superior vermian and cerebellar cortical veins with venous ectasia. However, there were no other venous congestions in the other vascular territories. Of note, an aneurysm with a maximum diameter of 3 mm was detected in the right ADS (Fig. 2). The fistula was diagnosed as Borden type 3 and Cognard type 4. Since the CT revealed a thick SAH in the posterior fossa and there were no other bleeding sources such as an aneurysm or vessel dissection, a feeder aneurysm was suspected as the source of bleeding.

**Fig. 3:**
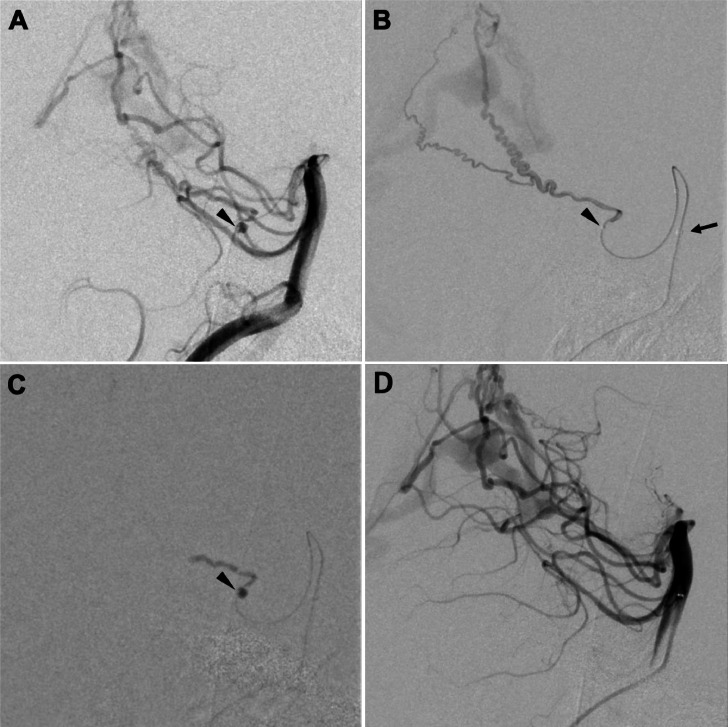
Zoomed lateral view of the angiogram (A) of the right vertebral artery with the edge of the microcatheter distal to the aneurysm (arrowhead). Lateral view of the angiogram (B) of the right ADS; the edge of the microcatheter is just distal to the aneurysm (arrowhead). Note that the distal access catheter is navigated to the upper basilar trunk (arrow). Lateral view of the NBCA cast (C) injected from the aneurysm to the middle of the ADS. Lateral view of the angiogram (D) of the vertebral artery revealing the disappearance of the right ADS and confirming the patency of the posterior cerebral artery.

On the second day after hospitalization, we performed embolization of the right ADS and aneurysm using NBCA under general anesthesia to prevent rebleeding from the aneurysm in the acute phase. A 90-cm 6Fr FUBUKI dilator kit (ASAHI INTECC, Aichi, Japan) was introduced into the right vertebral artery, and an Excelsior SL-10 microcatheter (Stryker, Kalamazoo, MI, USA) was navigated into the aneurysm at the right ADS with a CHIKAI 14 microguidewire (ASAHI INTECC, Aichi, Japan). To facilitate smooth navigation of the microcatheter, a Guidepost (Tokai Medical Products, Aichi, Japan) was navigated into the upper basilar trunk and used as the distal access catheter (Fig. 3A and 3C). A 33% NBCA–lipiodol mixture was injected into the right ADS and aneurysm (Fig. 3D). Subsequent angiography revealed the patency of the right PCA (Fig. 3B). Post-treatment CT demonstrated no signs of new bleeding.

One month after the SAH, we performed a second endovascular treatment to obliterate the fistula. Transarterial embolization through the left ADS and right MMA resulted in complete occlusion of the fistula. The procedure was performed without any complications. Magnetic resonance imaging performed after the procedure demonstrated no adverse events. The patient was transferred to a rehabilitation hospital with a modified Rankin Scale score of 4.

## Discussion

The annual incidence of intracranial and spinal cord dAVF is 0.29 per 100,000 population in Japan. Furthermore, tentorial dAVF is relatively rare and reported to be 2.9%-5.0% of all dAVFs [1,3]. There is no established treatment method for tentorial dAVF, and the treatment method is selected according to the location of the shunt point and the angioarchitectures in each case. Currently, when we choose embolic agents for cerebrovascular diseases, we have several options including coils, NBCA, polyvinyl alcohol particles, and liquid polymers (Onyx, ev3 Neurovascular, Irvine, CA USA). NBCA has a disadvantage of causing serious complications depending on the operator's handling, However, NBCA more effectively embolizes smaller lesions compared with other agents [8].

In this case, the ADSs were feeders to the fistula, and the feeder aneurysm was suspected to be the cause of the SAH. Due to the aneurysm's modest maximum diameter of approximately 3 mm and the marked curvature of the feeder beyond the distal neck, the selection of NBCA was favored over coil embolization. The rationale behind this decision stemmed from concerns that achieving complete occlusion of both the aneurysm and the right ADS using coils would have posed challenges. However, excessive NBCA backflow could lead to the occlusion of the right PCA. Therefore, we carefully injected the NBCA to avoid occluding the right PCA. Consequently, we completely occluded the feeder and the aneurysm while protecting the patency of the right PCA. Vascular displacement was minimized by elevating the intermediate catheter to a sufficient height, thereby reducing the risk of ADS dislocation during catheter removal [7]. Based on our case, NBCA can be an option for ADS aneurysm and feeder embolization.

To our knowledge, the association between ADS and dAVF is rarely reported. A systematic review of arterial aneurysms associated with intracranial dAVF reported that cerebral arterial aneurysms occurred in only 2 (6.4%) of 31 cases, except for arterial aneurysms on the surface of the dura mater [4]. In clinical practice, we sometimes observe SAH due to an aneurysm on the feeder of an arteriovenous malformation [5]. However, there are few reports of dAVF with arterial SAH, particularly in feeder aneurysms. Based on our literature review, there was only one case report of a dural AVF with a ruptured ADS aneurysm in the subarachnoid space treated with endovascular therapy with coiling [6].

In conclusion, we present the first case to underscore the safety and usefulness of NBCA embolization for a ruptured ADS aneurysm.

## Patient consent

The patient provided informed consent.
